# Technological advances in visualizing and rewiring microtubules during plant development

**DOI:** 10.1093/jxb/eraf284

**Published:** 2025-06-26

**Authors:** SungWoo Park, Andrew Muroyama

**Affiliations:** Department of Cell and Developmental Biology, Division of Biological Sciences, UC San Diego, 9500 Gilman Dr., La Jolla, CA 92093, USA; Department of Cell and Developmental Biology, Division of Biological Sciences, UC San Diego, 9500 Gilman Dr., La Jolla, CA 92093, USA; North Carolina State University, USA

**Keywords:** Cytoskeleton, methods, microtubule, optogenetics, plant development, super-resolution microscopy

## Abstract

Microtubules are crucial regulators of plant development and are organized by a suite of microtubule-associated proteins (MAPs) that can rapidly remodel the array in response to various cues. This complexity has inspired countless studies into microtubule function from the subcellular to tissue scale, revealing an ever-increasing number of microtubule-dependent processes. Developing a comprehensive understanding of how local microtubule configuration, dynamicity, and remodeling drive developmental progression requires new approaches to capture and alter microtubule behavior. In this review, we will introduce the technological advancements we believe are poised to transform the study of microtubules in plant cells. In particular, we focus on (1) advanced imaging and analysis methods to quantify microtubule organization and behavior, and (2) novel tools to target specific microtubule populations *in vivo*. By showcasing innovative methodologies developed in non-plant systems, we hope to motivate their increased adoption and raise awareness of possible means of adapting them for studying microtubules in plants.

## Teaser

In this review, we discuss the technological advances that are generating new insights into the organization and functions of microtubules in plant cells.

## Introduction

The microtubule cytoskeleton controls essential processes in the plant cell, from growth to chromosome segregation (Hsiao and Huang, 2023). Each cell maintains a microtubule network with characteristic organization that, nonetheless, must remain capable of rapid reorganization in response to developmental or environmental signals. Since microtubule functions are so varied, researchers have been eager to untangle the complex pathways both upstream and downstream of microtubule regulation. However, as microtubules are indispensable across the plant life cycle, many of the traditional ways of perturbing microtubules result in pleiotropic effects, complicating interpretation of associated developmental phenotypes. To overcome this major hurdle, new technologies have been developed to precisely image, quantify, and disrupt microtubule arrays in a cell type-specific manner with subcellular resolution. Here, we focus on the significant advances that have been made over the past decade in two specific areas—high-resolution, quantitative imaging methods and approaches to disrupt microtubules *in vivo*. As we do so, we introduce some of the key methods that are widely used to interrogate microtubules in plant cells and highlight the challenges associated with working with microtubules in plants. Finally, as we summarize some of the new biological insights revealed by using these methods in plants, we will discuss novel technologies that have been successfully deployed in non-plant species that could be ported into plant studies in the future.

## Technological challenges associated with visualizing microtubules in plant cells

**Fig. 1.**
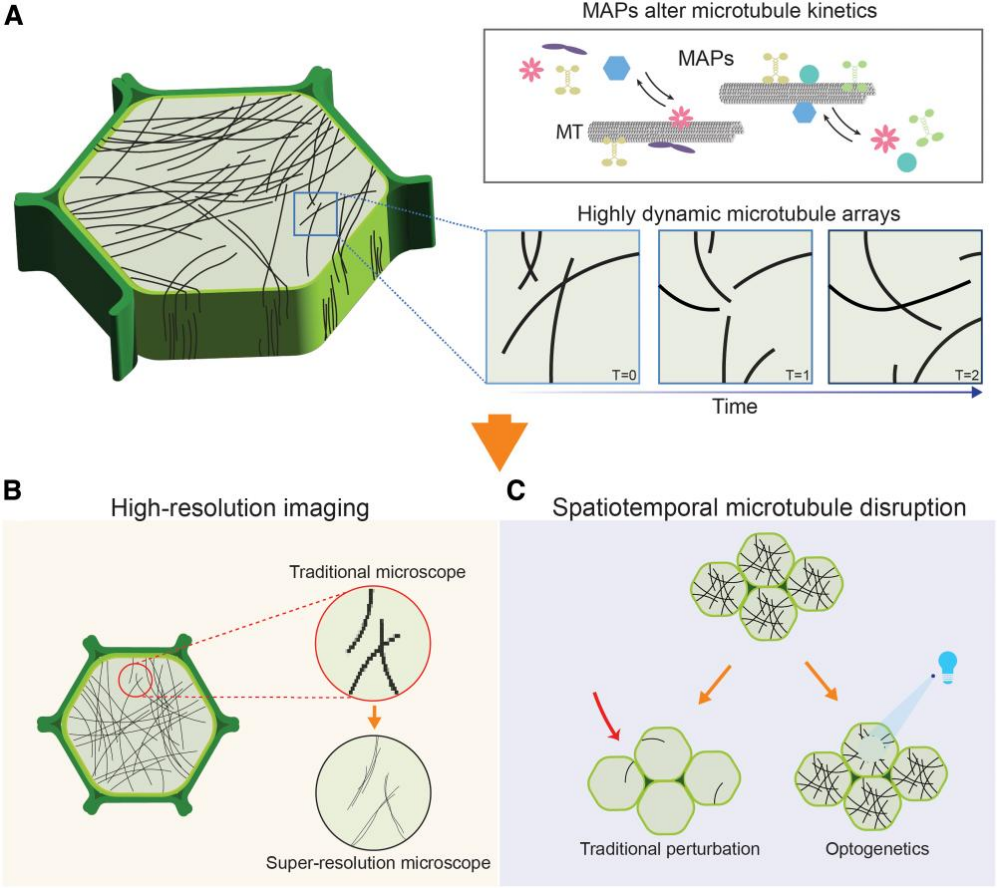
Challenges associated with studying microtubules, along with novel technologies that can overcome these obstacles. (A) Microtubule organization within a plant cell is complex and ever evolving. A wealth of microtubule-associated proteins (MAPs) are expressed in a cell type-specific and cell cycle-dependent manner to control microtubule kinetics. Together with the intrinsic instability of microtubules, MAP expression leads to highly dynamic microtubule arrays that are locally regulated and undergo rapid transitions, making it imperative to develop ways to capture them with high spatiotemporal resolution. (B) Super-resolution microscopy enables nanometer-scale visualization of microtubule networks, overcoming the resolution limits of conventional microscopy. (C) Genetically encoded microtubule disruption tools, such as optogenetics, allow for spatially and temporally precise manipulation of specific microtubule arrays in targeted cell populations, overcoming the pleiotropic effects of pharmacological inhibitors.

The first step toward understanding microtubule function within a given cell type is thorough characterization of the topology of the microtubule network, also known as the array. This is an inherently challenging problem. Microtubules are highly dynamic protein polymers composed of α/β tubulin heterodimers with an intrinsic polarity that governs differential behavior at each end of the filament (Horio and Murata, 2014). The picture is further complicated by the large number of microtubule-associated proteins (MAPs) that recognize specific features of microtubules and impact their kinetics (Hamada, 2014). Therefore, the geometry of the array at any given time is a function of the rates of nucleation, severing, stabilization, polymerization, and depolymerization of each filament, with substantial changes across the cell cycle and local variability along different cell faces (Fig. 1A).

Because microtubule arrays are in constant transition, the ideal experimental setup would allow researchers to capture time-resolved snapshots of each microtubule within the cell. Indeed, such optimally resolved images would be advantageous to our understanding of many microtubule-dependent processes in plants. For example, microtubule self-organization is thought to be an important driver of expansion-associated morphological changes, based, in part, on models of microtubule organization in cells of defined geometry (Mirabet *et al.*, 2018; Jacobs *et al.*, 2022). These models are capable of making predictions of how microtubule organization will evolve over time but currently rely on validation using incomplete data because of the challenges associated with tracking individual microtubules with high resolution over time in many cell types. Therefore, the ability to perform high-resolution, time-lapse imaging of microtubule arrays within specific cell populations would allow researchers to benchmark, validate, and refine these computational models, advancing our understanding of this important developmental process.

There are many challenges associated with imaging microtubules *in vivo*. While immunostaining has proven useful in specific cases, particularly in non-model species where transformation remains impossible, expensive, or otherwise technically challenging, it comes with limitations. Antibody delivery across the cell wall is difficult, and the danger of introducing fixation-induced artifacts means that most groups monitor microtubule localization using fluorescent proteins in transgenic lines when possible. These probes come in two basic flavors: those encoding a fluorescently tagged tubulin (TU) or a fluorescently tagged MAP. Fluorescently tagged tubulins (e.g. TUA5 and TUB6) are incorporated into polymerizing microtubules (Ueda *et al.*, 1999; Shaw *et al.*, 2003; Nakamura *et al.*, 2004), while the most widely used fluorescently tagged MAPs (e.g. mammalian MAP4 and MAP65–2) recognize and bind along the microtubule lattice (Marc *et al.*, 1998; Vavrdová *et al.*, 2020). Importantly, there are MAPs that recognize specific microtubule features, including the growing plus end of a tubule, (e.g. END BINDING 1, EB1) (Dixit *et al.*, 2006; Komaki *et al.*, 2010), the minus end (e.g. SPIRAL2) (Fan *et al.*, 2018; Nakamura *et al.*, 2018) or sites of microtubule nucleation (γ-tubulin ring complex components such as GCP2, GCP3 or GCP-WD) (Nakamura *et al.*, 2010; Walia *et al.*, 2014). The wealth of reporters currently available gives researchers the ability to pick one that highlights the microtubule feature they are interested in, for example using EB1-GFP to visualize growing plus ends that can be hard to resolve in a microtubule-dense cortex. Careful consideration of reporter choice is particularly important when deciding which image-analysis pipelines will eventually be used to quantify the data.

One of the major drawbacks of these reporters is that both types can hyper-stabilize, artificially bundle, or otherwise alter microtubule dynamics, sometimes dramatically. Expression level, therefore, is an important parameter, and care should be taken to: (1) Ensure that reporter expression does not impact development and (2) validate findings with multiple reporters when possible. Additional considerations include emission from the non-microtubule-associated cytosolic pool of the reporters, which can negatively impact the signal-to-noise ratio, and the difficulty of resolving microtubules at cell interfaces where plasma membranes are juxtaposed. Some of these issues can be alleviated by using ‘weaker’ or cell-type specific promoters, although there exists a non-trivial trade-off in signal intensity that can negatively impact resolution.

When imaging microtubules, researchers must also overcome challenges inherent to obtaining high-resolution images of plant cells, including significant autofluorescence from both intracellular components, such as chloroplasts and the cell wall, and sharp declines in fluorescence signal when imaging internal tissues owing to increased light scattering in deep samples. As such, microtubule arrays in epidermal cells, which are superficial, remain the most well-described and are often used for high-resolution characterization of microtubule behavior. Mounting strategies on both laser scanning and spinning disk confocal microscopes have been developed, enabling microtubule imaging in some particularly interesting cell types, including the cells in the L1 layer of the shoot apical meristem (SAM) and the stomatal lineage in developing leaves (Davies and Bergmann, 2014; Hamant *et al.*, 2019; Harline and Roeder, 2023). Imaging in high-interest cell types, such as those in the developing vasculature or those surrounding the quiescent center (QC) in the root meristem, remains particularly challenging when using confocal microscopy due to their depth from the organ surface, but some progress has been made by using cell type-specific reporters (Vilches Barro *et al.*, 2019; Zhang *et al.*, 2021; Stöckle *et al.*, 2022). Therefore, new means of visualizing and quantifying microtubule behavior are needed to overcome these long-standing limitations.

## Super-resolution imaging enables detailed characterization of microtubule arrays

Microtubules have a diameter of approximately 25 nm and are, therefore, under the diffraction limit (∼250 nm) of traditional light microscopes including widefield, confocal, and total internal reflection fluorescence (TIRF) systems. To overcome this barrier and enable the precise localization of smaller objects within biological samples, scientists have developed various super-resolution imaging techniques that have brought down the resolution to under 10 nm. Over the past several years, researchers have begun to apply these super-resolution approaches to the study of microtubules in plant cells, allowing for previously unattainable pictures of microtubule organization (Fig. 1B).

Structured illumination microscopy (SIM) is a technique that allows for an approximately two-fold improvement in resolution (∼100 nm) and works by imposing an illumination pattern during the excitation of the sample (Gustafsson, 2000). Several images are acquired as the pattern is shifted, from which a super-resolution image can be deconvolved. SIM can be used to resolve diffraction-limited objects within the cell, including plasmodesmata, traveling cellulose synthase particles, and meiotic chromosomes (Fitzgibbon *et al.*, 2010; Morgan and Wegel, 2020; Duncombe *et al.*, 2022). Several studies have applied SIM to the study of microtubule organization in the hypocotyl epidermis, allowing researchers to resolve individual microtubules in bundles, perform high-resolution tracking of plus-end behaviors, and compare preprophase band formation between Col-0 and *KATANIN1* mutants (Komis *et al.*, 2014; Vavrdová *et al.*, 2019, 2020). SIM can also be used to obtain detailed information about MAP behavior. For example, when SIM was used to image EB1-GFP-expressing hypocotyl cells in *Arabidopsis*, the size of the EB1 comet was shown to change as the rate of plus-end polymerization changed over time (Shaw *et al.*, 2019).

Resolution can be further enhanced by using techniques such as Stochastic Optical Reconstruction Microscopy (STORM) (Rust *et al.*, 2006; Huang *et al.*, 2008) and Photoactivated Localization Microscopy (PALM) (Betzig *et al.*, 2006). These conceptually similar imaging strategies create super-resolution images by computationally refining the position of individual fluorophores. To accomplish this, hundreds of images of a single sample are acquired, using specific wavelengths of light to sparsely and stochastically turn on and off individual fluorophores. The centers of the emitted light are fit with a point spread function, and the super-resolution image is constructed as the position of each fluorophore is identified. While this approach can resolve individual microtubules, it is generally not compatible with live cell-imaging because of the large number of images that must be acquired to build the super-resolution image. Nonetheless, because STORM requires the use of antibodies and photoswitchable dyes, it could, theoretically, be used on any plant species. Indeed, STORM has been successfully used in *Arabidopsis*, including to capture high-resolution (20–40 nm) images of microtubules in roots (Dong *et al.*, 2015; Maß *et al.*, 2020; Traeger *et al.*, 2025). However, the aforementioned challenges with immunostaining dynamic structures like microtubules in plant cells mean that PALM, which uses protein fusions to photoswitchable fluorophores, may be more accessible for super-resolution studies in genetically tractable species. Several studies have successfully used PALM to look at microtubule organization in the hypocotyl epidermis (Vavrdová *et al.*, 2020) and to reveal new insight into membrane protein dynamics (Bayle *et al.*, 2021). Moving forward, we are starting to see super-resolution techniques combined with other approaches such as expansion microscopy, where samples are embedded in a hydrogel and isotropically swelled to increase the distance between molecules. Recently, a couple of groups reported methodology for performing expansion microscopy in *Arabidopsis* roots and demonstrated that this approach increased the ability to resolve microtubule arrays (Gallei *et al.*, 2025; Grison *et al.*, 2025).

## Light-sheet microscopy enables rapid and gentle time-lapse imaging

Because microtubules are highly dynamic, an optimal imaging setup would allow very fast acquisition times with minimal photobleaching. Recently, several groups have shown how light-sheet microscopy can be used to produce videos of microtubule behavior with high spatiotemporal resolution. In contrast to scanning confocal microscopes, a light-sheet microscope illuminates the sample using a sheet of light in a defined focal plane (Chen *et al.*, 2014). The beam is scanned through the sample, enabling rapid acquisition times. Additionally, because the light sheet only excites fluorophores within the focal plane being imaged, photobleaching due to out of plane excitation is dramatically reduced, a major improvement over other imaging modes.

Light-sheet microscopy is particularly useful when studying plant development over time. For example, it is ideal for long-term tracking of cell behavior or high spatiotemporal resolution of rapid subcellular dynamics (Ovečka *et al.*, 2018; Schütz *et al.*, 2021; Sinclair *et al.*, 2024; Winter *et al.*, 2024; Lee *et al.*, 2025). Several studies have used systems to specifically look at microtubules. For example, Ovecka *et al*. used light-sheet microscopy to image the root meristems in 35S::GFP-TUA6 seedlings, allowing them to get improved resolution deep within the tissue (Ovečka *et al.*, 2020). The same group also performed light-sheet microscopy of preprophase bands and microtubule structures during mitosis in a *Medicago sativa* line expressing a fluorescent reporter utilizing the microtubule-binding domain (MBD) of MAP4 (35S::GFP-MBD), highlighting that the method can be used in other plant species besides *Arabidopsis* (Vyplelová *et al*., 2017).

## Machine-learning approaches for quantitative microtubule analysis

Clearly, there are myriad variables to consider when deciding how best to capture images of microtubules. There are just as many considerations to weigh when deciding how to conduct robust and reproducible image analyses. Fortunately, many groups have invested significant resources into these problems and have developed a host of freely available programs that semi-automate quantification of microtubule behavior and organization. Several tools, including the ImageJ/FIJI plugin FibrilTool, are routinely used and allow users to quantify the major features of microtubule networks, including plus-end dynamics, degree of anisotropy, and their major axis of orientation (Applegate *et al.*, 2011; Jacques *et al.*, 2013; Boudaoud *et al.*, 2014; Faulkner *et al.*, 2017). These software packages can be combined with others that can segment cells, such as MorphoGraphX (Strauss *et al.*, 2022), to obtain complementary information about cell expansion and proliferation. These approaches continue to be invaluable resources for the community, but new types of imaging data and a large number of analysis approaches based on machine learning suggest that a new wave of analysis tools are on the horizon.

Machine learning, in particular, has revolutionized the field of image analysis over the past several years, rapidly becoming just as, or more, accurate than human observers in picking out salient image features.

Two program classes hold particularly high potential to generate new biological insight into microtubule organization and behavior. The first are programs that can be used to reconstruct super-resolution images from lower resolution images or otherwise improve signal-to-noise within the sample, thereby improving the ability to resolve microtubules (Fang *et al.*, 2021; Kölln *et al.*, 2022; Chen *et al.*, 2023). The second are programs that can detect and segment microtubules or MAPs and report quantitative information, including microtubule density and plus-end behavior (Masoudi *et al.*, 2020; Nagao *et al.*, 2020; von Chamier *et al.*, 2021). Generally, these programs are trained to recognize specific image elements using a large sample of ground truth data. Generating and annotating the training data can be a rate-limiting step in model development, so some groups have started to explore the possibility of using generative models to create synthetic images of microtubules to improve model accuracy (Saguy *et al.*, 2025). Since the underlying classifiers in these deep learning programs often remain opaque and may be robust for only one type of imaging data, careful validation of new models and comparison to ground truth data is essential when exploring these new image analysis methods. This a rapidly evolving field, and there are now platforms that allow researchers without significant in-house expertise in generative AI models to utilize these tools for the analysis of subcellular dynamics (von Chamier *et al.*, 2021).

## Genetically encoded microtubule disruptors

While the involvement of microtubules in cellular processes during all life stages makes them a rich source for biological discovery, their developmental necessity creates a major challenge to isolating the functions of specific microtubule arrays *in vivo.* For decades, researchers have employed pharmacological inhibitors of microtubule dynamics to study the assembly, binding of MAPs, and developmental functions of microtubule arrays in plants (Morejohn and Fosket, 1991). These include chemical agents that either depolymerize or stabilize microtubules, such as oryzalin and paclitaxel, respectively. Because their pharmacokinetics have been extensively characterized and they can be used to perturb microtubule function across tissues and species, they remain very useful for many applications. However, new tools would provide complementary approaches to studying plant microtubules, and there has been a push over the past several years to develop and utilise new genetically encoded tools to perturb microtubules *in planta*. The major advantage of these is the ability to limit microtubule disruption to specific cellular populations. As described in more detail below, this has allowed researchers to study the developmental outcomes following microtubule disruption in a way that was previously inaccessible with pharmacological inhibitors, which cause pleiotropic effects upon sustained application.

What tools are available to disrupt microtubule organization within specific cellular populations? A strategy that has been effectively used in animal species (Muroyama and Lechler, 2017) and more recently in plants, revolves around cell type-specific overexpression of microtubule destabilizing proteins. In plants, overexpression of a phosphatase-inactive form of PROPYZAMIDE-HYPERSENSITIVE 1 (PHS1ΔP), a protein whose expression is typically induced by stresses like osmotic stress, rapidly destabilizes cortical microtubules (Fujita *et al.*, 2013). Driving expression using founder-cell or endodermal promoters and imaging during the process of lateral root initiation has revealed the respective contributions of cortical arrays in these cells to lateral root emergence (Vilches Barro *et al.*, 2019; Stöckle *et al.*, 2022). Recently, researchers created a line where the promoter of WUSCHEL-related homeobox 1 (WOX1) drives PHS1ΔP expression to destabilize microtubules at the tip of developing sepals, which negatively impacted cell growth and caused defects in floral development (Trinh *et al.*, 2024). In the future, the same principles could be applied to create cell type-specific constructs that affect other aspects of microtubule dynamics, including stabilization or polymerization rate. Driving transgene expression with inducible systems could, therefore, enable precise targeting of microtubule organization during specific developmental stages (Zuo *et al.*, 2000; Schürholz *et al.*, 2018).

## Optogenetics allows subcellular control of protein localization and activity

While these newer genetic approaches offer substantial advantages over traditional pharmacological or mutant analyses, they still lack the spatiotemporal precision required to study microtubule dynamics at the subcellular level. Fine-tuned manipulation of microtubule organization or protein recruitment to subsets of microtubules could be useful, for example, to study highly local effects of microtubules on cell shape, as these are proposed to drive cell lobing during pavement cell morphogenesis (Sampathkumar *et al.*, 2014; Lauster *et al.*, 2022), or specific microtubule populations during mitosis. Optogenetics, which utilizes light-sensitive protein domains to control protein activity, has emerged as a powerful tool to overcome these limitations. Researchers have taken advantage of natural and evolved variation in light-sensitive proteins from plants, yeast, and bacteria to develop a range of genetically encoded light-inducible protein tools. By combining recent advances in illumination technologies with increased availability of these light-sensitive domains, researchers have innovated new strategies that enable rapid, reversible, and micron scale-controlled perturbation of intracellular microtubule organization via optogenetics (Fig. 1C).

Light-inducible dimerization (LID) or oligomerization systems are among the most widely used optogenetic tools for regulating protein localization and activity. The basic strategy behind these systems is to control the association of two proteins by fusing one to a minimal domain from a photoreceptor that undergoes a light-induced conformational change, and fusing the other to the peptide that binds to the photoreceptor domain in either its ‘dark’ or ‘light’ state. Widely used examples include the cryptochrome 2 (CRY2) / Cryptochrome-Interacting Basic-Helix-Loop-Helix (CIB) protein pair from *Arabidopsis thaliana* (Kennedy *et al*., 2010) and the *Neurospora crassa* photoreceptor Vivid (VVD), which undergoes light-induced homodimerization (Zoltowski *et al.*, 2007; Kawano *et al.*, 2015). Dimerization pairs have now been developed that respond to different wavelengths of light and have vastly different on-off kinetics, allowing researchers to pick pairs that are well-suited for their biological question and system.

**Fig. 2.**
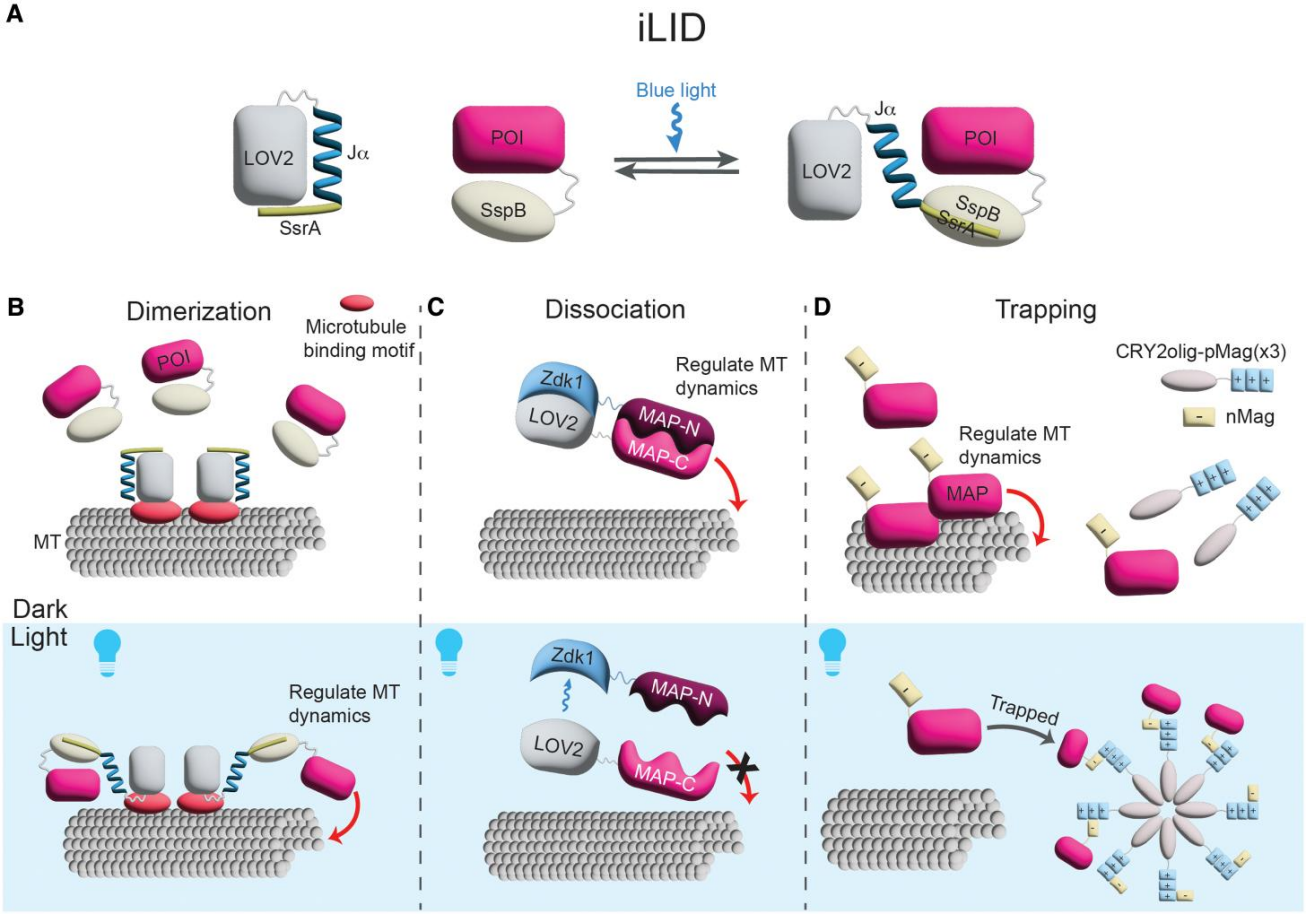
Optogenetic approaches to target and manipulate microtubules. (A) The improved light-inducible dimerization (iLID) system enables reversible photodimerization upon blue light exposure. The Light-Oxygen-Voltage2 (LOV2) domain undergoes a conformational change in response to blue light, allowing the SsrA peptide to bind its partner, SspB, which can be fused to a protein of interest (POI). (B) The iLID system can be adapted to recruit POIs to microtubule subpopulations upon blue light exposure by fusing a microtubule-binding motif to the LOV2 domain and SspB to the POI. (C) Combining the Zdark1 (Zdk1)-LOV2 system with a split MAP allows researchers to control MAP activity using light. (D) A light-inducible oligomerization complex can be used to sequester MAPs upon blue light exposure, allowing control over MAP localization and activity.

One of the most popular systems takes advantage of the Light-Oxygen-Voltage2 (LOV2) domain from *Avena sativa* phototropin 1, which undergoes a well-characterized conformational change where the C-terminal Jα helix undocks from the LOV2 domain upon blue light exposure (Fig. 2A). Improved light-inducible dimerization (iLID) system is a ‘light-on’ system that exploits the inherent photochemical properties of the LOV2 domain by incorporating a short SsrA peptide, originally derived from *E. coli*, within the Jα helix of the LOV2 domain (Guntas *et al.*, 2015). In the absence of light, this SsrA sequence cannot bind its partner, SspB. However, the SsrA sequence becomes exposed upon illumination with blue light, allowing it to bind SspB. In this way, protein association can be spatiotemporally controlled by generating two protein fusions, one with the LOV2-SsrA module and the other with SspB, and selectively illuminating a region of the cell or tissue (Fig. 2B). Wang *et al*., extended the utility of LOV2 domain-fusions by developing LOV2 Trap and Release of Protein (LOVTRAP), which utilizes Zdark1 (Zdk1), a small peptide engineered from the Z domain of Staphylococcal protein A that selectively binds to the LOV2 domain in the dark but dissociates upon blue light illumination (Wang *et al.*, 2016). This system, therefore, is a complement to iLID that allows light-induced protein-protein dissociation.

## Optogenetic strategies to target microtubules and MAP activity

Several groups have developed novel ways to use these light-sensitive protein pairs to both titrate MAP activity and locally control association with microtubules at specific sites within the cell. Kevin Slep and colleagues developed an optogenetic tool utilizing the iLID system to achieve temporally controlled recruitment of proteins to microtubule plus ends in an end-binding protein-dependent manner (Adikes *et al.*, 2018). To target iLID to microtubule plus ends, they fused the iLID module to the EB-binding SxIP motif to confer plus-end tracking to the iLID module through its interaction with EB proteins. The protein of interest was then fused with SspB, allowing light-dependent recruitment to microtubule plus ends. By illuminating *Drosophila* S2 cells co-expressing the SxIP-iLID construct and tgRFP-SspB with blue light, they could show that blue light stimulation triggers rapid colocalization of tgRFP-SspB and SxIP-iLID at microtubule plus ends within 1.5 seconds, effectively providing a means to target desired proteins to growing microtubule plus ends. Importantly, recruitment was fully reversible upon light removal, highlighting the power of this strategy. Leveraging this tool, the authors further investigated the impact of actin–microtubule crosslinking on microtubule dynamics by fusing SspB to actin-binding domains and demonstrated that F-actin cross-linking slows down microtubule growth.

Light can also be used to position MAP activity. Opto-Katanin is a two-part system that targets the microtubule-severing protein katanin to locally disassemble both dynamic and stable microtubules upon blue-light illumination (Meiring *et al.*, 2022). The first component is a fusion between iLID, the N-terminus of EB3 (EB3N), which has low affinity for the microtubule lattice as a monomer, and VVDfast, a blue light-sensitive homodimerization domain that exhibits rapid reversibility. Upon blue light exposure, VVDfast dimerizes, enhancing EB3N's microtubule lattice binding affinity. The second component is a fusion between the p60 subunit of katanin and SspB. The two-component strategy effectively minimized dark-state activity, allowing efficient light-induced microtubule-targeting and hexamerization of p60 to induce local microtubule disassembly with micron-scale resolution.

A study from van Haren *et al*. developed a clever optogenetic tool that allows EB1 activity to be removed upon illumination (van Haren *et al.*, 2018) (Fig. 2C). To accomplish this, they created π-EB1, a split form of the growing plus-end tracking protein EB1 where the EB1 N-terminus was fused to LOV2 and the EB1 C-terminus was fused to Zdk1. The authors showed that the two-part π-EB1 could restore endogenous EB1 function in the dark and that the protein would inactivate rapidly upon illumination. The authors used this system to demonstrate that local EB1 dissociation could be used to steer cancer cell migration *in vitro*. This same approach could potentially be applied to other MAPs if functional split forms can be developed, opening numerous avenues for future applications in microtubule research.

An alternate approach to control protein activity is to reversibly trap proteins into large, inactive complexes using light (Lee *et al.*, 2014) (Fig. 2D). This strategy has been used to develop a novel light-inducible clustering system called OptoTrap to disrupt microtubules and inhibit kinesin-1 activity *in vivo* (Y. Xu *et al.*, 2024). OptoTrap consists of two blue light-responsive components: CRY2olig and Magnets. CRY2olig is a mutant version of CRY2 that exhibits enhanced light-induced clustering (Taslimi *et al.*, 2014). Magnets come in two flavors—positive magnet (pMag) and negative magnet (nMag)—that are engineered from VVD to selectively heterodimerize with each other through electrostatic interactions only under light illumination (Kawano *et al.*, 2015). In OptoTrap, CRY2olig is tagged with three copies of pMag and the protein of interest is fused with nMag. Upon blue light illumination, CRY2olig-pMag(3X) forms large protein clusters that recruit and sequester nMag and, therefore, the protein of interest. The authors showed that α-tubulin and kinesin-1 puncta formed in seconds to minutes after blue light exposure. Puncta dissociated within minutes to hours after being returned to the dark, demonstrating that OptoTrap enables rapid, reversible protein clustering at subcellular resolution. Excitingly, this system is modular and modifiable, allowing researchers to tune aggregate size and alter dissociation kinetics to suit their own needs.

## Extending optical control of microtubules

Optogenetic tools can be combined with other technologies to expand our ability to study microtubule dynamics in greater detail. A recent example from Van Geel *et al*. combined Förster resonance energy transfer (FRET) with optogenetics to simultaneously manipulate local microtubule dynamics and measure free tubulin concentrations (Van Geel *et al.*, 2020). To monitor free tubulin concentration, the researchers used a previously developed stathmin FRET sensor that reports relative amounts of free tubulin in the cytosol (Niethammer *et al.*, 2004). Binding to tubulin triggers stathmin to adopt a stiff, elongated conformation, increasing the distance between fluorophores and reducing FRET efficiency. The researchers combined this FRET sensor with a photoactivatable form of Ras-related C3 botulinum toxin substrate Rac1 (PARac1) that can bind its effectors upon blue-light illumination (Wu *et al.*, 2009). Rac1 activation leads to stathmin phosphorylation, which disrupts its ability to bind to tubulin heterodimers (Wittmann *et al.*, 2004). By combining these two systems, the researchers could locally alter the availability of free tubulin using PARac1 and simultaneously monitor the relative amount of free tubulin using the FRET sensor. This clever merging of existing sensors and probes highlights the measurements and perturbations that can performed *in vivo* by combining optogenetics with other probe varieties.

Optochemical tools offer a complementary means of manipulating the cell by activating and deactivating chemical compounds using light. These offer a distinct advantage over optogenetic approaches in that they are theoretically compatible with any cell type/species with a tubulin sequence that is sufficiently homologous to human tubulin sequences, which were used to create these compounds. One example is photostatin—a photoactivatable analog of combretastatin A-4, which binds at the colchicine binding domain in β-tubulin and prevents α/β heterodimers from adopting the straight configuration needed to form microtubules (Lu *et al.*, 2012; Borowiak *et al.*, 2015). Photostatins were shown to be potent and fully reversible microtubule depolymerizers in human and mouse cells and could be used to disrupt mitosis within a single cell in a developing *C. elegans* zygote. The range of these compounds has since been extended to include stabilizers (Müller-Deku *et al.*, 2020; Gao *et al.*, 2022), further expanding their possible uses. While experimental tests would be required to assess cell wall permeability, these compounds could be useful for a variety of studies in plants and be readily deployed outside of *Arabidopsis*.

## Current challenges associated with applying optogenetics in plants

Optogenetic tools, including but not limited to those mentioned above, offer immense potential for spatiotemporal control of microtubule dynamics. However, applying these techniques to plants presents inherent challenges. Plants rely on ambient light for energy via photosynthesis, and growing plants in the dark dramatically alters their growth and development (Konrad *et al.*, 2023; Wu *et al.*, 2024). Since most optogenetic constructs are activated by wavelengths found under standard plant growth conditions, workarounds are required to prevent unintended activation of optogenetic switches. Additionally, plants express photoreceptors that may become aberrantly activated or otherwise interact with the optogenetic constructs, many of which are designed using protein domains from these same receptors. Therefore, carefully considered experimental design is paramount for successful optogenetic applications in plants (Konrad *et al.*, 2023; Beyer and Ramírez, 2024).

Researchers are actively exploring ways to surmount these challenges. One notable example is the Plant Usable Light-Switch Elements (PULSE) system, which uses two light-activated constructs with opposing activity to change transcriptional output based on whether the plant is exposed to white light or to monochromatic red light (Ochoa-Fernandez *et al.*, 2020). The first component of this system is B_off_, a fusion between a LOV domain from *Erythrobacter litoralis* EL222 and a transcriptional repressor domain that forms dimers upon blue-light illumination, triggering DNA binding and transcriptional repression. The second component is R_on_, a PhyB-PIF6 red-light-inducible split transcription factor switch (Müller *et al.*, 2014), where DNA-localized PIF6_(aa1-100)_ recruits PhyB fused to a VP16 activation domain and activates transcription under red light (660 nm). In the dark, neither B_off_ nor R_on_ affect transcription. Under white light, both B_off_ and R_on_ are activated, but B_off_ dominates and transcription is repressed. Only under monochromatic red light is R_on_ selectively activated to induce transcription. This innovative strategy using multiple, stacked circuits highlights how synthetic biology approaches can enable optogenetics in plants.

Beyond PULSE, researchers are exploring additional strategies to expand optogenetic applications in plants, including using non-plant-derived photoreceptors or harnessing the green-yellow spectrum. Because plant photoreceptors primarily absorb UV, blue, red, and infrared light, the ability to shift activation to the green-yellow spectrum would be advantageous. While the green-yellow spectrum remains largely untapped for optogenetics thus far, some groups have made strides in this area. For example, Chatelle *et al*. engineered CarH, a transcription factor activated by green light (Chatelle *et al.*, 2018) and Piccini *et al*. developed orange carotenoid proteins (OCPs) that respond to blue-green light (Piccinini *et al.*, 2022). For some studies looking at cellular level phenotypes, it may be possible to utilize transient expression and experimental synchronization with the normal light-dark cycle that the plants are typically grown under, although this strategy is unlikely to succeed in revealing long-term developmental effects.

These innovative strategies illustrate some of the new, creative solutions for overcoming the challenges of optogenetic applications in plants. While most existing optogenetic constructs in plants are limited to regulating transcriptional activity or ion channels, ongoing advancements create hope that optogenetic tools capable of modulating microtubules at the subcellular level in plants could be developed in the near future.

## Conclusions and future prospects

We believe that the study of microtubules in plant cells has reached a transition point where the technologies highlighted here will begin to be more widely adopted. The cost of commercial microscopes capable of new imaging modes remains high for individual labs, but light-sheet and super-resolution-capable microscopes are now routinely found in shared microscopy facilities, vastly improving access. However, it is important to remain mindful of the fact that most commercial systems were designed for applications in animal cells and, as such, significant experimental optimization is likely required to image plant samples. Therefore, we applaud the continued publication of methods papers detailing both microscopy successes and failures, which are a particularly valuable resource for labs that do not have extensive in-house imaging expertise.

In the same vein, reports describing new programs for improved image reconstruction, classification, and quantification are highly valuable to the community. The widespread and continued use of programs like FibrilTool (Boudaoud *et al.*, 2014) and MorphoGraphX (Strauss *et al.*, 2022) speak to the enthusiasm for user-friendly and robust analysis tools. In response to this, programs like ZeroCostDL4Mic have been developed to allow users to input their own training data to create customized models (von Chamier *et al.*, 2021). These tools are essential steps to make quantitative image analysis at scale feasible, especially as dataset sizes are predicted to grow as higher resolution images are acquired over longer time frames.

In parallel to improvements in imaging technology, novel microtubule probes and ways to introduce them into plants will be essential to expanding the research toolkit. Groups that create robust methods for monitoring or disrupting microtubules *in vivo* have the opportunity to introduce transformative technologies, as evidenced by the rapid adoption of PHS1ΔP (Vilches Barro *et al.*, 2019; Vaddepalli *et al.*, 2021; Mosca *et al.*, 2024; Trinh *et al.*, 2024). In particular, we are closely watching the new probes that report on more subtle microtubule features, including tubulin isoform usage, post-translational modifications, and microtubule lattice expansion. These, and other, microtubule characteristics create significant microtubule heterogeneity within a single cell and inspired the idea of a complex ‘tubulin code’ that can be written and read by the cell to add additional layers of control to microtubule-dependent processes (Roll-Mecak, 2020). Much of this complexity has yet to be studied in plants, although there are tantalizing hints that some features of this tubulin code may be important for plant development (Gardiner, 2019). Several new fluorescent reporters have been created that are (1) capable of reading out these microtubule characteristics, and (2) compatible with live-cell imaging (Jansen *et al.*, 2023; K. Xu *et al.*, 2024; Zhou *et al.*, 2024). Thus far, they have been used exclusively in animal cells, but tools like these which report on conserved features between plant and animal tubulins are potentially transferrable to diverse plant species.

Finally, recent improvements to genome editing in plants open wide the potential avenues for future microtubule research. Tissue-specific CRISPR knockout (CRISPR-TSKO) of MAPs will help to elucidate cell type-specific consequences of microtubule disruption that can be indecipherable in full knockouts (Decaestecker *et al.*, 2019). New CRISPR/Cas9 constructs have been created that finally allow the generation of in-frame fluorophore insertions at native genomic loci (Miki *et al.*, 2018; Li *et al.*, 2024), which will help mitigate some of the overexpression-induced artifacts common to microtubule reporter lines. At the same time, CRISPR is making it possible to generate fluorescent reporters in non-model plant species (Veley *et al.*, 2021), creating new possibilities for comparative studies of microtubule functions across the plant kingdom and opening the door to interrogate microtubule-dependent developmental processes found only within certain branches of the green lineage.
